# A clinical registry of dementia based on the principle of epidemiological surveillance

**DOI:** 10.1186/1471-2377-9-5

**Published:** 2009-01-28

**Authors:** Josep Garre-Olmo, Margarita Flaqué, Jordi Gich, Teresa Osuna Pulido, Josefina Turbau, Natalia Vallmajo, Marta Viñas, Secundí López-Pousa, Registry of Dementia of Girona Study Group (ReDeGi Group)

**Affiliations:** 1 Research Unit, Institut d'Assistència Sanitària, Salt, Spain; 2 Dementia Unit, Hospital de Palamós, Palamós, Spain; 3 Neurodegenerative Disease Unit, Hospital Universitari Josep Trueta, Girona, Spain; 4 Neurology Department, Hospital de Figueres, Figueres, Spain; 5 Neurology Department, Hospital de Campdevànol, Campdevànol, Spain; 6 Geriatrics Department, Hospital d'Olot, Olot, Spain; 7 Neurology Department, Hospital de Blanes, Blanes, Spain; 8 Dementia Unit, Hospital de Santa Caterina, Salt, Spain

## Abstract

**Background:**

Traditional epidemiological studies do not allow elucidating the reality of referral and diagnosis patterns of dementia in routine clinical practice within a defined territory. This information is useful and necessary in order to plan and allocate healthcare resources. This paper presents the results from a dementia case registry based on epidemiological surveillance fundamentals.

**Methods:**

Standardised registry of dementia diagnoses made in 2007 by specialised care centres in the Health Region of Girona (RSG) (Spain), which encompasses an area of 5,517 sq. km and a reference population of 690,207 inhabitants.

**Results:**

577 cases of dementia were registered, of which 60.7% corresponded to cases of Alzheimer's disease. Presenile dementia accounted for 9.3% of the cases. Mean time between the onset of symptoms and clinical diagnosis was 2.4 years and the severity of the dementia was mild in 60.7% of the cases. High blood pressure, a family history of dementia, dislipidemia, and a past history of depression were the most common conditions prior to the onset of the disease (>20%).

**Conclusion:**

The ReDeGi is a viable epidemiological surveillance device that provides information about the clinical and demographic characteristics of patients diagnosed with dementia in a defined geographical area.

## Background

Epidemiology research in the area of dementia has been carried out on the basis of cross-sectional observational studies and longitudinal cohort studies that have established the prevalence, incidence and mortality rates of the main subtypes of dementia. [1] These studies have also made it possible to identify risk factors and open new research lines. [2,3] Nevertheless, this methodological approximation, due to its high economic cost and the logistic complexity that the follow-up of participants overlong periods of time represents, provides limited information on the impact of the dementia cases on the healthcare system in terms of needs and utilisation of resources. [4] Moreover, this type of studies does not allow to elucidate what the reality of detection, referral and diagnosis is in routine clinical practice of primary care services and specialised care in a specific territory. However, given the progressive ageing of the population, this information is essential for proper planning and allocation of health and social care services in the territory, in accordance with patient needs at mid and long term. Other epidemiological strategies such as a clinical registry of cases in a delimited geographical area could be a complimentary approach.

The creation of registries for certain diseases is standard procedure in some pathologies such as cancer or cardiovascular diseases but uncommon in the area of dementias certain. A number of initiatives do exist however such as the Consortium to Establish a Registry for Alzheimer's Disease (CERAD) [5] and others. [6-8] Nevertheless, these initiatives, of variable duration, have mainly been geared towards the creation of cooperative networks, the primary objective of which, by means of clinical procedures and standardised diagnoses, is to increase the statistical power of the clinical research. At present, there are only two dementia registries for which references can be found in the specialised literature [9-12], although these registries have non-comparable methodologies and registry scopes.

The objective of this paper is to present descriptive data for 2007 from the Registry of Dementia of Girona (ReDeGi), a clinical registry of dementia cases based on the guidelines of epidemiological surveillance. [13]

## Methods

### Design

Consecutive and standardised registry of new dementia cases in a delimited geographical area.

### Geographical area of reference and population under surveillance

**Figure 1:**
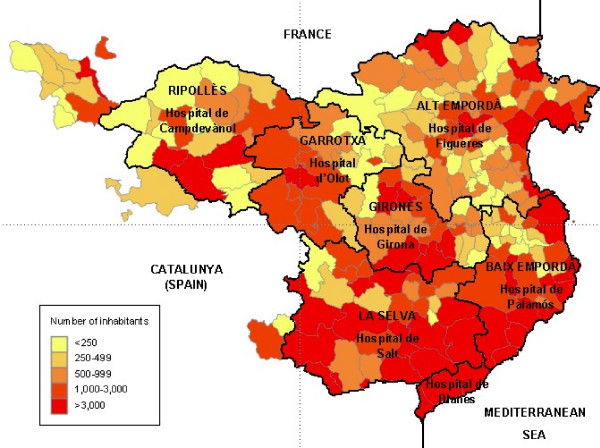
**Number of inhabitants per municipality in the catchment area of RSG hospitals**.

The Health Region of Girona (RSG) is located in the north-east of Catalonia (Spain). It has an extension of 5,517 km^2^, a population of 690,207 inhabitants (according to data from the Citizens Municipal Registry 2007) and a population density of 125.1 inhabitants/km^2^. The RSG includes 221 municipalities, 91 of which (43.5%) have 500 or less inhabitants, representing 4.3% of the population. Eighty per cent of the municipalities have 3,000 or less inhabitants and only eight municipalities have over 20,000 registered inhabitants. The RSG is composed of seven healthcare sectors that virtually coincide with the political-administrative division of the territory into 7 boroughs (Alt Empordà, Baix Empordà, Garrotxa, Gironès, Pla de l'Estany, Ripollès and Selva). The RSG provides 6 borough hospitals (healthcare level I) and a reference university hospital (healthcare levels I and II), with each hospital having an allocated reference population. Figure 1 shows the municipalities that make up the catchment area of each hospital and the number of inhabitants stratified into large groups. The ReDeGi keeps up a registry of new cases of dementia diagnosed in the 7 RSG hospitals.

### Case registry

The ReDeGi fulfil the Centers for Disease Control (CDC) guidelines for evaluating a surveillance system [13]: it controls an assigned population under surveillance and provides standardised criteria for case definition and a simple and flexible system for data collection. The methodological principles and the functional structure of the ReDeGi have been previously described. [14,15] In brief, the ReDeGi registers all new cases of dementia diagnosed in hospitals pertaining to the RSG, in accordance with the clinical diagnosis criteria established for dementia syndrome and the different subtypes according to the Diagnostic and Statistical Manual of Mental Disorders (DSM-IV-TR). [16] In a complementary fashion, clinical research criteria are used. [17-23] Diagnoses may have been made in the neurology or geriatrics outpatient consultation offices of each hospital. Case identification and notification to the ReDeGi is performed by the actual consultants of each RSG hospital that conducted the diagnostic process of dementia. A technician from the ReDeGi regularly visits the RSG hospitals and reviews the medical records of the dementia cases notified, writing the information in a 4-section data collection form: 1- identification of the centre (name of the hospital, date of admission and medical record number); 2- sociodemographics (date of birth, sex, nationality, place of residence, work position, schooling level, marital status, type of housing and healthcare referral device); 3- diagnosis characteristics (approximate date of the onset of symptoms, date of the diagnosis, DSM-IV-TR diagnostic criteria, additional diagnostic criteria for the subtype of dementia); 4- clinical data (score and date of administration of the Mini-Mental State Examination (MMSE) [24], score and date of administration of the Blessed Dementia Rating Scale (BDRS) [25], score of the Clinical Dementia Rating (CDR) [26], a past family history of dementia, present diagnosis of hypertension, diabetes mellitus, dislipidemia, stroke, thyroid disease and a past history of depressive disorder). The collected information is then entered into an electronic database that meets the confidentiality requirements for personal data protection in compliance with Spanish legislation.

### Diagnosis of dementia

The validity of the diagnosis is based on the premise of validity of the diagnostic process on the part of the specialist physicians collaborating with the ReDeGi. Specialist physicians from each hospital (specialists in neurology or geriatrics) performed the registered diagnoses of dementia. Diagnosis was established on the basis of the medical history, an interview the patient and a close observer (family member or carer), a general medical examination, results from laboratory tests (haematology and basic biochemistry tests) and diagnostic neuroimaging (computerised axial tomography and/or brain magnetic resonance) when needed. The age of onset of symptoms was determined within the context of the interview with the informant during the diagnosis process. During this interview the specialist physician ask the informant about the approximate date of the onset of symptoms.

### Statistical analysis

A descriptive analysis of the variables was carried out using central tendency and dispersion measures for quantitative variables and absolute and relative frequency measures for qualitative measures. The relative frequencies for the entered diagnoses of dementia were calculated and stratified by sex and the severity of the dementia. Bivariate statistical analysis was carried out to determine the existence of significant differences according to the sex and the severity of the dementia by applying parametric and non-parametric techniques, according to the distribution of data. [27]

To estimate the diagnostic coverage in the registry's catchment area, a dementia prevalence of 5.4% and an incidence of 8.8 cases were assumed per 1,000 people per year at risk for individuals aged 60 and over. These values were estimated by a group of international experts using a Delphi consensus. [28] Based on the available evidence from multiples epidemiological studies carried out all over the world, this report provides a consensus about the frequency indicators of dementia around the world. Specifically, the rates used in this study correspond to the geographic area known as EURO A, which includes Western European countries. Data for the population in the catchment are of the registry correspond to those published by the National Institute of Statistics for the referred area. [29] Results are expressed as absolute numbers and percentages, median (range) and mean (standard deviation) values, and 95% confidence intervals (CI). A statistical significance level of 95% was set for hypothesis contrasts.

## Results

### Viability of the data collected

Most of the information required by the ReDeGi was documented in the medical chart of the patients, which also showed a low frequency of lost values in the 28 variables. The variable that presented the highest number of missing values was the approximate date of onset of the symptoms which was missing in 12.3% (n = 71) of cases, followed by the schooling level accounting for 11.1% (n = 64), the presence of a past family history of dementia with 9.5% (n = 55), healthcare referral device accounting for 7.3% (n = 42), type of housing arrangement 6.1% (n = 35), CDR score 3.6% (n = 21), MMSE score 2.8% (n = 16) and date of administration of the MMSE accounting for 2.1% (n = 12).

### Subtypes of dementia

Over the course of 2007, 577 cases of dementia were entered, of which 60.0% were cases of Alzheimer's disease (AD), 10.7% were cases of mixed dementia (AD + vascular component), 4.7% were cases of vascular dementia (VaD), 26.6% corresponded to other subtypes of dementia, and 8.7% to unspecified dementia. The frequency of presenile dementia with an onset of symptoms before the age of 66 was 9.3%; mean age in these cases was 58.9 years (SD = 7.1; Range = 32–64). Table 1 presents the absolute and relative frequencies of the dementia diagnoses entered following the DSM-IV-TR diagnostic criteria. Primary care centres referred 75.2% of the cases, 10.1% were referred by a hospital medical department, 2.8% by the Mental Health Network and the remaining 4.7% by long-term care centres and other private means of healthcare.

**Table 1:** Absolute and relative frequency of dementia diagnoses included in the registry according to DSM-IV-TR criteria

	**n (%)**
	
***Alzheimer's type dementia ***[F00]	
***- early non-complicated onset ***[F00.00]	7 (1.2)
***- early onset with a depressed mood ***[F00.03]	2 (0.3)
***- late non-complicated onset ***[F00.10]	255 (44.2)
***- late onset with delusional ideation ***[F00.11]	38 (6.7)
***- late onset with a depressed mood ***[F00.13]	44 (7.7)
***Vascular dementia ***[F01]	
***- non-complicated ***[F01.80]	18 (3.1)
***- with delusional ideation ***[F01.81]	2 (0.3)
***- with a depressed mood ***[F01.83]	7 (1.2)
***Dementia caused by Pick's disease ***[F02.0]	23 (4.0)
***Dementia associated with Parkinson's disease ***[F02.3]	15 (2.7)
***Alcohol-induced persistent dementia ***[F10.73]	1 (0.1)
***Dementia caused by non-specified diseases ***[F02.8]	
***- non-specified***	14 (2.5)
***- Lewy's bodies***	32 (5.6)
***- head injury ***	1 (0.1)
***- corticobasal degeneration***	1 (0.1)
***- supranuclear progressive palsy***	4 (0.7)
***- multiple aetiologies (Alzheimer's + vascular component)***	62 (10.7)
***Dementia caused by Creutzfeldt-Jakob's diseases ***[F02.1]	1 (0.1)
***Non-specified dementia ***[F03]	50 (8.7)

### Demographic and clinical characteristics

Mean age was 78.9 years (SD = 7.8; Range = 33–95) and 62.6% were women. Mean age for males was lower than that of females (76.9 years vs. 80.3 years; p < 0.001). 60.7% of the cases were mild dementia, 26.5% were moderate and 9.2% severe. As regards the education, 21.3% were illiterate or had never attended school, 60.1% had between 1 and 8 years of schooling and only 7.4% had 9 or more years of schooling. 45.2% of the patients were married or had a live-in partner, 42.3% were widowers or widows. There were also significant differences regarding the marital status (13.9% of widowed men as compared to 59.3% of widowed women; p < 0.001). The place of residence was their own home in 59.4% of the cases, the residence of a family member in 28.2% of the cases and only 6.6% of the cases lived in a nursing home. The percentage of patients who lived in their own home was greater in men (82.7% vs. 52.2%) whom also showed a lower percentage of nursing home residence than women (3.6% vs. 8.4%; p < 0.001).

Mean time of progression from the onset of the first symptoms to the time of diagnosis was 2.4 years (SD = 1.8; Range = 0.1–10.3), with no differences regarding patient gender (p = 0.059). Mean MMSE and BDRS scores were 16.8 (SD = 5.4; Range = 0–29) and 7.9 (SD = 4.3; Range = 0.5–22) respectively. A lower MMSE score was observed in women (16.2 vs. 18.1; p < 0.001) and a greater score in the BDRS cognitive subscale (3.8 vs. 3.2; p < 0.001) as well as in the BDRS functional scale (1.4 vs. 0.9; p = 0.016), but not so in the behavioural disturbances subscale (3.0 vs. 3.3; p = 0.086).

In 25.1% (n = 145) there was a past family history of first-level dementia (29.2% in males and 26.9% in women; p = 0.567), 50.6% of cases had been diagnosed with high blood pressure (42.1% in males and 56.5% in females; p = 0.001), 17.3% had been diagnosed with DM (n = 100) (19.9% in males and 16.1% in females; p = 0.240), in 25.1% there was a diagnosis of dislipedimia (21.3% in males and 27.8% in females; p = 0.083), in 14.0% (n = 81) there was a past history of CVA or stroke (19.0% in males and 11.2% in females; p = 0.010), in 5% a diagnosis of thyroid disease (2.3% in males and 5.9% in females; p = 0.045), in 21.3% (n = 123) a past history of depression (16.7% in males and 24.5% in females; p = 0.027). Table 2 shows the main clinical and demographic characteristics stratified according to the severity of the dementia in accordance with the CDR score.

**Table 2:** Clinical and sociodemographic characteristics stratified according to CDR*

	**CDR 1**	**CDR 2**	**CDR 3**
	
**Age**, *mean (SD)*	77.5 (7.6)	81.4 (6.5)	83.0 (7.1)
**Females**, *n (%)*	207 (59.3)	102 (66.7)	40 (75.5)
**Schooling**, *n (%)*			
***Illiterate/not attending school***	68 (21.3)	38 (27.7)	16 (34.8)
***Between 1 and 8 years***	220 (69.2)	91 (66.4)	27 (58.7)
***9 and over***	30 (9.5)	8 (5.9)	3 (6.5)
**Marital status**, *n (%)*			
***Single/separated***	12 (3.7)	9 (6.2)	6 (12.3)
***Married/partner***	183 (56.7)	55 (38.2)	13 (26.5)
***Widower***	128 (39.6)	80 (55.6)	30 (61.2)
**Residence**, *n (%)*			
***Own home***	241 (74.6)	74 (49.3)	17 (32.7)
***Home of family member/other***	75 (23.2)	61 (40.7)	25 (48.1)
***Nursing Home***	7 (2.2)	15 (10.0)	10 (19.2)
**Referral device**, *n (%)*			
***Primary Care***	268 (76.8)	119 (77.8)	37 (69.8)
***Others***	81 (23.2)	34 (22.2)	16 (30.2)
**Years of evolution**, *mean (SD)*	1.9 (1.5)	2.6 (1.7)	3.1 (2.4)
**MMSE**, *mean (SD)*	19.1 (4.5)	14.0 (4.5)	10.5 (5.3)
**BDRS**, *mean (SD)*			
***Cognitive function***	2.8 (1.2)	4.6 (1.4)	5.7 (1.5)
***Functional capacity***	0.2 (1.5)	2.0 (1.5)	4.9 (2.3)
***Behaviour***	2.7 (1.7)	3.7 (2.0)	3.0 (1.8)
**Past family history of dementia**, *n (%)*	102 (31.5)	27 (19.9)	13 (27.7)
**Diagnosis of high blood pressure**, *n (%)*	166 (47.7)	86 (56.6)	32 (62.7)
**Diagnosis of diabetes mellitus**, *n (%)*	57 (16.4)	27 (17.9)	11 (21.6)
**Diagnosis of dislipidemia**, *n (%)*	91 (26.1)	32 (21.1)	16 (31.4)
**Diagnosis of stroke**, *n (%)*	38 (10.9)	30 (19.9)	7 (13.7)
**Diagnosis of thyroid disease**, *n (%)*	16 (4.6)	8 (5.3)	1 (2.0)
**Past history of depression**, *n (%)*	86 (24.7)	27 (17.9)	5 (9.8)

* Results for available data; CDR: Clinical Dementia Rating; MMSE: Mini-Mental State Examination; BDRS: Blessed Dementia Rating Scale

### Diagnostic coverage in the catchment area

In accordance with the 2007 Municipal Census Registry, the RSG population embraced 690,207 inhabitants, of which, 15.8% were over the age of 65. In accordance with these data and based on the estimated prevalence (5.4%), the number of prevailing cases of dementia in the territory amounted to 6.034, with 103.433 people over the age of 65 being at risk of developing dementia in 2007. In accordance with the incidence rate used, (8.8/1,000), the estimated number of new dementia cases for the territory in 2007 was 910 cases. Taking into account that out of the 577 entered cases of dementia 570 corresponded to RSG inhabitants, the registry's coverage embraced 61.3% (CI 95% = 59.4–65.8) of the cases. Table 3 shows data pertaining to the registry's coverage for the catchment area of each hospital within the RSG region. The raw incidence of new dementia diagnosis in the RSG during 2007 was 5.5/1.000.

**Table 3:** Catchment population and diagnostic coverage according to the health sector within the RSG

**Geographical catchment area**	**Catchment area**	**>64 years (%)**	**Estimated number of prevalent cases**	**Estimated number of incident cases**	**Registered cases **(%)
***H. Girona***	141,924	20,585 (14.5)	1,132	171	120 (70.2)
***H. Salt***	133,983	20,182 (15.1)	1,110	168	131 (78.0)
***H. Olot***	53,187	10,942 (20.6)	602	91	40 (44.0)
***H. Figueres***	131,556	21,730 (16,5)	1,195	181	69 (38.1)
***H. Blanes***	79,027	9,794 (12,4)	539	81	80 (98.8)
***H. Palamós***	124,192	20,059 (16,2)	1,103	167	72 (43.1)
***H. Campdevànol***	26,338	6,175 (23.4)	340	51	58 (100.0)

Total*	690,207	109,467 (15.9)	6,034	910	570 (61.3)

* 7 cases entered corresponded to patients domiciled outside the RSG area

## Discussion

Dementia is a disease that fulfils the parameters proposed by the CDC in order to assess the importance and need for an epidemiological surveillance system in terms of public health: frequency and severity, differences and dissimilarities amongst population groups, high cost, potential of primary and secondary prevention, and public interest. [13] The ReDeGi sets out from a conceptual model of registry, which fulfil the CDC guidelines for evaluating a surveillance system and is adapted to the particular needs of dementia. [14] Following a 2-year long test in which the operating structure of a registry system took the lead with satisfactory results [15], 2007 represented the start of activity of the ReDeGi in the entire RSG area which extends for 5,517 km^2 ^and includes a population of 690,207 inhabitants. The results obtained are an innovation with regard to the existing dementia registries and can be evaluated from a point of view of methodology or from a practical utility perspective.

On the one hand, from a strictly methodology perspective, the ReDeGi has adopted a registry model different to those used by the South Carolina Alzheimer's Registry and by the New York State Dementias Registry. The first of these is based on the integration of computerised data originating from different sources of information. The information entered includes the diagnosis of dementia (ICD-9 criteria), other medical diagnoses and basic demographic data. The information that enables the elaboration of a registry is circumscribed to the stratification of dementia subtypes according to demographic and administrative characteristics such as gender, race or age, and to the origin of the particular case. Access to death certificates via a link also allows estimating the mortality rates of entered cases from a certain inclusion date in the registry onwards. [11,12,30] Even though the New York State Dementias Registry only admits cases of dementia (ICD-9 criteria) from discharge forms from all the hospitals in the state of New York, it does allow to carry out an estimate of the hospitalisations required by patients with dementia and their causes as well as the type of admission and destination at discharge. [9,10] Unlike the previously mentioned registries, which are based on information from electronic databases, the ReDeGi is characterised by the compilation of data straight from the patient's medical chart by a ReDeGi technician. This method allows the standardised registration of clinical information available in the patient's medical history. Moreover, it is a procedure that permits the maintenance of a dementia subtypes registry in accordance with DSM-IV criteria and other diagnostic criteria specific to the subtypes of dementia that ICD-9 and administrative databases are unable to register. For example, in table 1 cases with the diagnostic category "Dementia caused by non-specified diseases" include a subcategory named "Lewy's bodies" that correspond to those cases that accomplished the diagnostic criteria for dementia with Lewy bodies, and the 23 cases under "dementia caused by Pick's disease" diagnostic category correspond to 18 cases that accomplished the diagnostic criteria for frontotemporal dementia and 5 cases the diagnostic criteria for primary progressive aphasia. In this sense, the data collection procedure implemented at the ReDeGI ensures greater validity of the information recorded in the registry related to the diagnosis.

On the other hand, from a strictly pragmatic point of view, the data obtained from the ReDeGi provide relevant information about the healthcare pressure brought about emerging dementia cases in a certain territory over a defined period of time. The ReDeGi provides evidence on the distribution of the dementia subtypes and the clinical and demographic characteristics of patients who contact specialist healthcare devices.

The analysis of the data shows that AD accounted for 60.1% of the entered cases, mixed dementia (AD + vascular component) for 10.7% and vascular dementia (VaD) for 4.6%. These percentages are similar to those from the South Carolina Alzheimer's Disease Registry, in which the AD cases entered in the period 1988–2003 accounted for 64% of the total and VaD cases accounted for the remaining 14%. In relation to the place of residence of the patients, the differences between the South Carolina Alzheimer's Disease Registry and the ReDeGi can reflect two distinct sociocultural and healthcare models. In the former model, 57% of the cases live in the community and 38% in assisted centres, whereas in the latter 83.1% live in the community (their own home or the home of a family member) and 6.6% live in long-term care centres. Data on the mean time between the onset of symptoms and clinical diagnosis indicate that for most dementias the mean time from onset to diagnosis is approximately 2 years. In this sense, it is hard to establish a precise date of dementia onset, although the estimate based on the data collected by the informant carer has proven to be a reliable source of information. [31]

The diagnostic coverage of the registry has been estimated to be 61.3% of the total expected cases in the territory, with differences being observed amongst the different healthcare sectors. However, there are certain limitations that should be taken into account when estimating the diagnostic coverage on the basis of expected number of dementia cases per year in the catchment area. Formally, in an epidemiological study, the incidence rate would be calculated dividing the number of cases detected in specified period by the free-of-disease inhabitants by time unit. However, the cases entered in the registry correspond to patients who were referred to a specialised diagnostic unit, some of whom presented moderate to severe severity and a mean time of 2 years from onset of symptoms to time of diagnosis. These are new cases of dementia for healthcare devices and consequently the results of the estimated diagnostic coverage are not representative of the real incidence in the general population. These results can however be reliably used to ascertain the incidence of new cases that contact specialised health services. A possible explanation for the number of cases not registered (38.7%) could be hypothetized that they correspond to mild or advanced cases of dementia. It may be that the first group did not attend specialised consultations due to the low impact of the symptoms of dementia in the early stages and the second group did not attend consultations because the benefit of a diagnostic could be more limited. It is interesting to note that we have observed a great variation in the diagnostic coverage across hospitals. These differences might due to different variables and may help to identify factors related to the referral and diagnosis in clinical practice. For example, these differences may be due to differences between types of specialist physicians (neurology or geriatrics) or due to the existence of specific devices for dementia diagnostic. However, it is important to emphasise that, for the ReDeGi, more than achieve a full diagnostic coverage, it is more important to register all cases that have been diagnosed in the 7 RSG hospitals. Thus, the ReDeGi will become a powerful information device in few years when it provides cumulative information about the pressure of dementia cases and its evolutionary course.

## Conclusion

The ReDeGi illustrates the application of epidemiologic surveillance to the setting of a chronic condition such as dementia and for which the available information usually comes from such dispersed sources as vital statistics, health surveys, hospital discharges, etc.

The ReDeGi is a valid, representative and updated source of information of great utility for the planning and allocation of the health and social care services needed by patients with dementia and of great special interest to healthcare administrators. The main limitation is that actually, the ReDeGi can't provide dementia incidence rates for its geographical area. However, as the ReDeGi continues registering new dementia diagnosis, in few years it will be possible to identify all cases with a reported onset during each calendar year, and to obtain population rates by age for variables of interest, such as rates of illiteracy or other. Future plans of the ReDeGi encompass an extension of the influence area of primary care devices at short term and an annual follow-up of the cases included in the registry. The proposed annual follow-up of registered cases will allow us to gain further insight into the course of the disease and to ascertain the percentage of cases that continue to live at their homes, the number of cases that are admitted into long-term care centres and the number of cases that die, as well as the clinical and demographic variables associated with such changes. The maintenance and extension of the registry over the next few years will allow us to describe how the pressure on healthcare services evolves and to determine the clinical features and course of the cases diagnosed with the disease. Although actually mild cognitive impairment (MCI) is a controversial diagnostic category [32], a potential application of the ReDeGi in the future may be to register cases with this diagnostic category from the neurology or geriatrics outpatient consultation offices using the same methodology. However, due to the low validity and reliability of the actual diagnostic criteria for MCI, this possibility will be considered in the future. An other potential application of the ReDeGi may be to conduct etiologic case-control studies by selecting comparable control subjects from primary care databases, to determine, for example, the odds of dementia between diabetic and non-diabetic patients. Moreover, this application will contribute to a better interpretation of the clinical characteristics of dementia patients respect the general population.

## Competing interests

The authors declare that they have no competing interests. The funding source had not involvement in the decision to submit the paper for publication.

## Authors' contributions

JGO performed the statistical analyses and drafted the manuscript. All authors (MF, JG, TOP, JT, NV, MV, SLP) edited and approved the final manuscript.

## Pre-publication history

The pre-publication history for this paper can be accessed here:


